# Is Abbot cast still relevant today? Preliminary randomized study based on 3D analysis

**DOI:** 10.1186/1748-7161-7-S1-O40

**Published:** 2012-01-27

**Authors:** J Deceuninck, JC Bernard, C Lecante, E Berthonnaud, S Verlet

**Affiliations:** 1 Croix Rouge française-CMCR des Massues Lyon, France; 2 Ets Lecante, Lyon, France

## Background

In 1913, it’s the first time we talk about Pr. Abbott for the correction of “old scoliosis”. Instructions for Abbott cast are:

· Stiff scoliosis with moderate to significant Cobb angle

· No cardio-pulmonary contraindication

· No cervico-occipital disorder

· No vessel fragility

But is Abbott cast still relevant today? And if it’s right, is it in the same conditions?

## Materials and methods

This is a preliminary randomized study lead at the CMCR des Massues in Lyon since April 2010 to January 2011 about 40 teenagers treated by Abbott cast. First group (n=17) have had 3D analysis before cast and second (n=23) group after cast. With the 3D analysis we have worked out if the thoracic band was put by classical method or was conversely put (based on plane of maximum curvature (PMC) parameters). We have analyzed Cobb angles in frontal and sagittal plane and PMC parameters.

## Results

There was no more cervical lordosis in 21, 7% in the second group (3D analysis after cast) and 5,8% in first group. There was an improvement of lumbar lordosis (10, 67°) in first group with a normalization of the number of vertebra in this one. There was lesser decrease of kyphosis in the first group but this result was not significant.

## Conclusions

Abbott cast is still relevant today but not with the same practical details. We have to introduce 3D analysis in our daily practice.

